# Assessment of First Aid Knowledge at Different Stages of Education

**DOI:** 10.3390/healthcare13131507

**Published:** 2025-06-24

**Authors:** José Ángel García-Blaya, J. Arturo Abraldes, Raquel Vaquero-Cristóbal

**Affiliations:** Research Group Movement Sciences and Sport (MS&SPORT), Department of Physical Activity and Sport, Faculty of Sport Sciences, University of Murcia, 30720 Murcia, Spain; joseangel.garcia5@um.es (J.Á.G.-B.); raquel.vaquero@um.es (R.V.-C.)

**Keywords:** lifeguarding, training, accident, safety, emergency

## Abstract

Background: Previous studies have pointed out the importance of having first aid knowledge to effectively tend to the victim of any accident. However, it is still a constant challenge to ascertain the level of first aid knowledge among students at different stages of education. Objectives: This study aimed to analyse differences in first aid knowledge and meaningful knowledge across stages of education, as well as the influence of gender, and to determine variations in the perceived importance of first aid by stage of education. Methods: To achieve this, the First Aid Knowledge Assessment Questionnaire was administered to a sample of 1088 students: 405 from compulsory secondary education, 298 from baccalaureate and vocational training, and 385 at the university level. Results: Both when the sample was analysed in general and when it was divided according to gender, significant differences according to level of education were found for all the analysed dimensions in both knowledge level and meaningful knowledge level (*p* = 0.010–<0.001; ES: 0.025–0.397), with university students generally showing the highest results and secondary school students the lowest, both in knowledge and meaningful knowledge (*p* = 0.040–<0.001). Secondary school students showed a significantly lower interest and knowledge in first aid and regarded it as less important for their personal and professional growth (*p* < 0.001). In contrast, university students rated first aid as highly important and showed a greater interest on the topic (*p* < 0.001). Conclusions: Differences were observed across academic levels in knowledge and meaningful knowledge in first aid, with university students scoring higher and being the ones who gave most importance to this content. The findings underscore the need to reinforce first aid education at all levels of education.

## 1. Introduction

Accidents represent a challenge in modern society, and first aid must be applied to prevent further injury or loss of life [1]. Road traffic accidents are one of the biggest problems worldwide, due to their high incidence and mortality rate [2]; heart attacks are also one of the leading causes of death in the European Union, as well as injuries at home, where more than 50% are suffered by people over 75 years of age, representing a high and increasing mortality [3]. Given these statistics, it is essential to have specific knowledge and skills to help the injured [4]. If the right action is taken, the damage can be minimised and/or the life of the injured person can be saved. For example, acting promptly would reduce the death rate in road traffic accidents to 33%, with most fatalities occurring within half an hour from the impact [5].

It is usually family and friends who are near to the injured person at the time of the accident, regardless of the type. Their immediate action and/or role as alert-givers for professional help is essential [6]. Unfortunately, numerous studies indicate that the level of knowledge in first aid is insufficient in many areas, reducing the options of the pop-ulation to appropriately react to an accident [7]. In this regard, 33.6% of young people report that acquaintances, relatives, and friends are deficient in resolving first aid situations [8]. Previous studies also suggest that adolescents have gaps in knowledge regarding different blocks of content related to frequent domestic accidents [9]. Therefore, first aid content should be instilled in the general population because of its simplicity and relevance to life.

In this context, the 2021 European Resuscitation Council Guidelines reinforce the need for immediate bystander response, emphasising early intervention with cardiopulmonary resuscitation (CPR) and automated external defibrillator (AED) use as key elements in the chain of survival. These guidelines highlight the importance of population-wide training in basic life support (BLS) as a public health priority [10]. In the same vein, the latest recommendations by the World Health Organization stress the importance of community-based first aid education as a strategy to improve the response capacity in emergency situations. These recommendations advocate empowering citizens and reducing morbidity and mortality from common emergencies [11].

First aid is a specific subject where specific training is key [12]. As a consequence of the above, some institutions provide training programmes that aim to train the general population in first aid. Research studies indicate that from the age of 11, basic first aid contents and skills can be mastered to provide adequate help in the event of an accident. As a result, compulsory secondary education in Spain, legislated in the Spanish context by the Spanish Ministry of Education, includes first aid contents in its education plan, ranging from prevention of to intervention in accidents. More specifically, in Spain, Organic Law 3/2020, of 29 December, which amends Organic Law 2/2006, of 3 May, on Education (LOE), establishes that in compulsory secondary education (CSE) first aid content must be taught, especially within the subject of physical education [13]. More specifically, in the first two years of this education stage, the first aid contents acquired in the primary education stage should be reinforced, while, in the last two years of CSE, more complex contents should be taught, with more autonomy for the students [14]. In the Region of Murcia, Decree 235/2022 of 7 December establishes that, in the first year of CSE, the Protect, Alert, and Rescue behaviour and the 112 protocol must be taught; in the second year of CSE, basic life support; in the third year of CSE, specific techniques such as the Heimlich manoeuvre; and, in the fourth year of CSE, resuscitation using AEDs and CPR. Thus, basic protocols, general knowledge, and simple and specific techniques should be mastered by the end of this stage of education [15].

A greater depth in these contents is acquired in later stages such as the baccalaureate (BAC) and vocational education and training (VET) stages or university degrees, mainly when the specialisation is in areas related to health and physical-sports activity [16]. In this sense, in the baccalaureate, Royal Decree 243/2022 of 5 April establishes the minimum order and teachings for this educational stage. The students begin the BAC stage knowing how to identify a cardiac arrest, call emergency services, start CPR, use the defibrillator, and resolve choking [17]. In VET, specific training courses regulated by Order EDU/90/2022 of 26 April and Royal Decree 570/2023 of 4 July include modules on emergency care, primary and secondary care, first aid techniques, basic life support, use of the AED, and actions in the event of haemorrhages and require a combination of theoretical and practical methods [18]. At the university level, the European Higher Education Area, governed in Spain by Spanish Organic Law 6/2001 on universities, establishes specialised first aid contents in areas related to health, exercise, and sport sciences [19]. Moreover, first aid contents are also included in the curricula of bachelor’s degrees in the field of health, exercise, and sport sciences [20,21]. Specifically in the Degree in Sports Science, the Resolution of 18 September 2018, of the General Secretariat of Universities, which publishes the Agreement of the Council of Universities of 17 September 2018, regulates these contents at the national level, stating that first aid is part of the specific compulsory training, as a descriptor of the module of physical activity and physical exercise for health and with special populations [22].

Despite the above, different studies have indicated that the level of knowledge in first aid is insufficient at different levels of the education sector [23,24]. Insufficient knowledge of first aid in secondary school influences the level of first aid in the following stages of BAC, VET, and/or university, making it necessary to reinforce the contents supposedly learnt during the different educational stages, in order to achieve the meaningful learning of these contents [25]. Problems persist also among VET students, where, despite the positive perception of self-knowledge by students, the level of competence is still insufficient [26].

Furthermore, it is not only important to act quickly but also in an appropriate way in this context [12]. This is because acting incorrectly in certain situations can even worsen the accident victim’s health situation. Therefore, it is just as dangerous, if not more dangerous, to have erroneous knowledge of what to do than not having a clear understanding of how to act and deciding not to act, because, by acting incorrectly, the situation will worsen [27,28]. Therefore, it is important for the general population to have meaningful knowledge on how to act appropriately in this type of stressful situations, in order for them to act and to do so in the right way [29].

However, no previous studies are known to have analysed whether the progressive structure of in-depth study of first aid content theoretically proposed by the Spanish Ministry of Education for the different stages of education leads to differences in the knowledge acquired in these subjects among students at different stages of education. On the other hand, previous studies have pointed out that women tend to receive more training on first aid than men [30], and show a significantly higher knowledge on how to provide first aid to an unconscious person [31]. However, no previous studies have analysed the gender differences in first aid knowledge at these stages of education or of the importance of having meaningful knowledge along the correct line in order to be able to tend to casualties properly and not to fall into the trap of making their situation even worse.

Therefore, the objectives of the present research were as follows: (1) to analyse the differences in knowledge and meaningful knowledge on first aid according to stage of education, as well as the influence of gender; and (2) to determine the differences in the importance given to first aid according to stage of education. The hypotheses of the present research were as follows: (1) the level of knowledge in first aid should be higher and more consistent as the student acquires higher levels of education, regardless of gender; and (2) students in higher stages of education should place more importance on first aid knowledge than students in lower stages of education.

## 2. Materials and Methods

### 2.1. Design

A cross-sectional study design was used, with a non-probabilistic convenience sample. The design was approved prior to initiation by the ethics committee of the University of Murcia (code ID: 3840/2022), following the declaration of Helsinki, and in accordance with the code of the world medical association. In addition, the research design and protocol were established according to the recommendations of the STROBE declaration [32].

### 2.2. Participants

The calculations necessary to establish the sample size were performed with Rstudio 3.15.0 software (Rstudio Inc., Boston, MA, USA). The significance level was set at α = 0.05. The standard deviation (SD) was established based on the final score obtained in the first aid questionnaire from previous studies (SD = 0.75) [33]. With an error (d) of 0.08 in the final score obtained in the first aid questionnaire, the required sample was 298 subjects per group, with this study aiming for a statistical power greater than 0.80 and achieving a calculated power of 0.96, which is considered high.

A total of 1088 adolescents and adults between the ages of 12 and 40 years old took part in this study (females: *n* = 345, mean age = 17.1 ± 4.81 years-old; males: *n* = 743, mean age = 18.3 ± 4.39 years-old). The participants were divided into three groups, one consisting of compulsory secondary education (*n* = 405; 122 females and 283 males), another consisting of baccalaureate (BAC) and vocational training (VET) students (*n* = 298; 117 females and 181 males), and a third consisting of university students (*n* = 385; 106 females and 279 males). The BAC and VET students were analysed in the same group, following previous research [34], as both stages of education were post-compulsory but pre-university studies.

The procedure consisted of speaking with the school administration to explain the research proposal, followed by informing the parents to obtain informed consent. Afterward, the students were informed about the research topic before applying the instrument to address any possible doubts. The inclusion criteria were a) being students at the institution in the specific stage of education and b), for vocational training and university degree students, being enrolled in a sports-related programme that included specific first aid training in the curriculum. The exclusion criteria were a) having an illness that would prevent them from completing the first aid questionnaire, such as an intellectual disability, and b), for vocational training and university degree students, having already completed the first aid courses included in the curriculum.

### 2.3. Instruments

All the participants self-completed the “Questionnaire for Assessing Knowledge Level in First Aid” [35]. The questionnaire can be found in Supplementary Material File S1. This questionnaire, which was validated, was endorsed by a panel of experts consisting of doctors, emergency technicians, and nurses. This panel of experts carried out a content validity check, reaching a consensus of over 80% on each of the items. Subsequently, a construct validation was performed by factor analysis, which resulted in the dimensions basic actions, wounds, trauma, allergies, CPR, and AED. Finally, the reliability of the instrument was analysed using an internal consistency analysis which showed a total Cronbach’s alpha of α = 0.784 and between α = 0.711 and 0.769 for the individual dimensions. The calculation of the Cronbach’s alpha of the meaningful knowledge showed a result of α = 0.778, with Cronbach’s alpha scores for the different dimensions between 0.722 and 0.793 [35]. The selection of institutions focused on public educational centres located in the Region of Murcia, as was the case in other studies [36,37].

The instrument was structured in two parts. The first part included demographic questions aimed at collecting sample data related to gender, age, and level of education. It also covered aspects such as interest in first aid content, the importance given to the subject, both in personal training and in the profession, and the current level of knowledge. These questions were presented using a four-point Likert scale (low, moderately low, moderately high, and high).

The second part consisted of 30 questions to assess knowledge on first aid, distributed across six dimensions: Basic Actions, Wounds, Trauma, Allergies, CPR, and AED, with five questions per dimension. A four-option Likert-type scale was used to answer these questions, assigning 2 points for the optimal response, 1 point for the correct response, 0 points for the neutral option, and −1 point for the incorrect response, based on the degree of accuracy in the proposed interventions, following the methodology of previous studies [35]. In addition, these negative scores were adopted because when validating the questionnaire, the expert panel understood that performing the actions included under this score could not only not benefit the injured person but be detrimental [35]. Once the data were collected, an analysis of the responses was carried out, organising them by content blocks. The results were expressed on a numerical scale ranging from 0 to 10. Subsequently, this numerical value was coded into categories based on the criteria established in previous research, so that a score between 0 and 3 reflected no knowledge; between 3 and 5, critical knowledge; between 5 and 7, minimal knowledge; between 7 and 9, adequate knowledge; and above 9, optimal knowledge [38].

Following previous research [33,39,40], the meaningful knowledge for all the questionnaire’s dimensions were also calculated. To this end, for each question to assess knowledge on first aid, the participants had to assign a score using a four-option Likert-type scale based on their level of confidence when answering. The options were as follows: very uncertain, somewhat certain, fairly certain, and very certain. Thus, to calculate the meaningful knowledge score for the dimensions Basic Actions, Wounds, Trauma, Allergies, CPR, and AED [20,26,27], the previously described methodology was applied by incorporating a corrective factor based on the confidence level expressed in the response [41]. The correction factors were ×1 for very uncertain, ×2 for somewhat certain, ×3 for certain, and ×4 for very certain.

### 2.4. Procedure

The data collection was carried out in various educational institutions located in the Region of Murcia. For the selection of these institutions, the schools in the region that offered secondary education, BAC, VET, or university degrees related to sports with first aid training were contacted. Data collection was performed in those institutions that voluntarily agreed to participate. Surveys were collected from a sufficient number of secondary, BAC, VET, and university institutions to ensure homogeneous groups in this study. The questionnaire was completed digitally by the participants through the University of Murcia’s survey platform (https://encuestas.um.es/encuestas/MjQ0NDU.c, accessed on 15 November 2024). To facilitate self-completion, students were taken to the Plumier classroom in their respective educational institutions, where they completed the surveys in a single session. The participants took approximately 25 min to complete the questionnaire.

All the participants completed the questionnaire under the same conditions. The researchers only assisted participants with general questions regarding the platform’s functionality or the generic operation of the questionnaire but did not help them complete it to avoid influencing their responses. When analysing the results, questionnaires completed in less than 10 min were discarded, as this was considered an insufficient amount of time for proper completion, following the methodology of previous studies [33,39,40].

### 2.5. Data Analysis

The normality and homogeneity of all study variables were verified using the Kolmogorov–Smirnov test. The Mann–Whitney U test and the Wilcoxon W test were used as test statistics. Descriptive statistics, including mean and standard deviation, were calculated for the quantitative variables, while frequency and percentage were calculated for the qualitative variables. An analysis of variance (ANOVA) was performed to analyse the differences in each dimension based on the level of education for the general sample. Subsequently, an analysis of covariance (ANCOVA) was conducted with gender as a covariate, along with an ANOVA with data segmentation by gender, to examine the influence of gender on the differences found in each of the dimensions analysed according to level of education.

To determine the presence of significant differences between groups for each variable, Bonferroni’s pairwise comparison was employed. Effect size (ES) was calculated using partial eta squared (η^2^), with ES values categorised as small (ES ≥ 0.10), moderate (ES ≥ 0.30), large (ES ≥ 1.2), or very large (ES ≥ 2.0), with the statistical significance set at *p* < 0.05 [42]. The χ^2^ test was used to analyse differences in interest, perceived importance, and self-perception of first aid knowledge according to level of education. Cramer’s V test (V) was employed to assess the magnitude of the association between variables and was classified based on previous research as follows: V < 0.17 (small magnitude), V > 0.17 and V < 0.29 (medium magnitude), and V ≥ 0.29 (large magnitude) [43]. The statistical significance for all the tests was set at *p* < 0.05. The statistical analysis was conducted using the SPSS statistical package (v.25.0; SPSS Inc., Chicago, IL, USA).

## 3. Results

Table 1 shows the differences in knowledge and meaningful knowledge of first aid according to level of education, including the influence of the covariate gender. Significant differences according to level of education were found for all the analysed dimensions in both knowledge level (*p* < 0.001; ES: 0.038–0.397) and meaningful knowledge level (*p* < 0.001; ES: 0.025–0.293), with a low to moderate effect size. The covariate gender showed a significant influence on both knowledge and meaningful knowledge of CPR and AED and in the total score for knowledge (*p* = 0.023–<0.001; ES: 0.005–0.010), with a low effect size.

**Figure 1:**
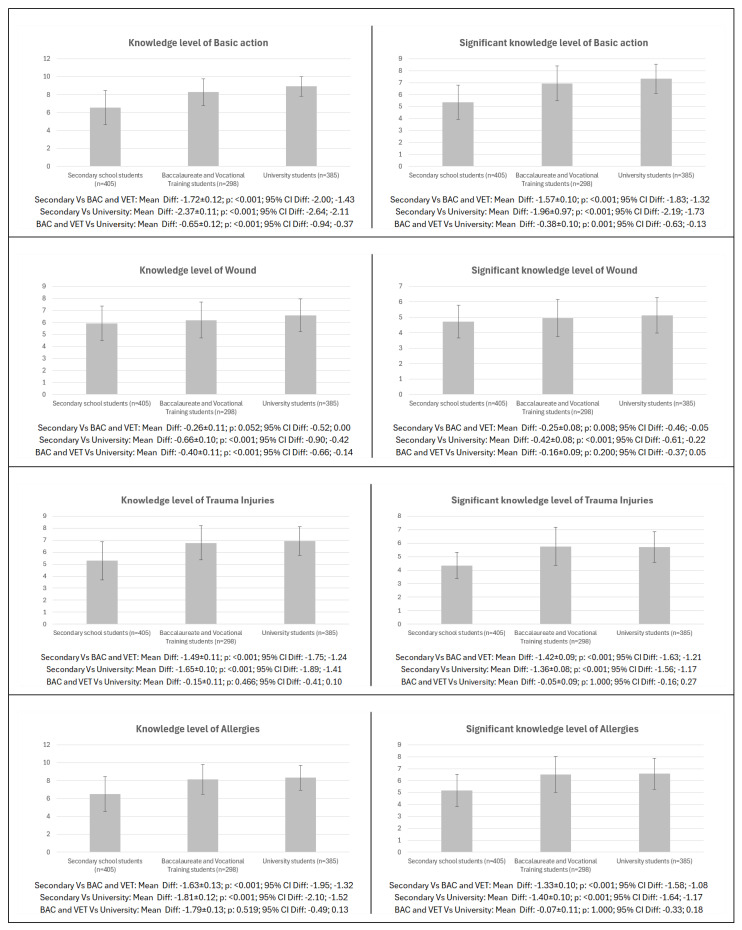

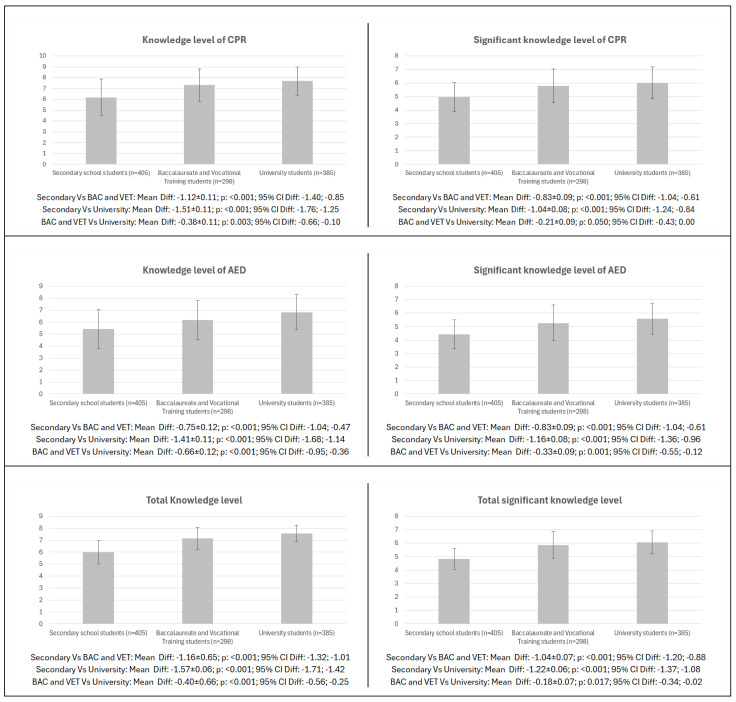
Pairwise differences in first aid knowledge and meaningful knowledge by level of education in the general sample. Legend: CPR: cardiopulmonary resuscitation; AED: automated external defibrillator; BAC: baccalaureate; VET: vocational training.

The pairwise comparison according to level of education for the different dimensions of knowledge and meaningful knowledge level is shown in Figure 1. Significant differences in knowledge and meaningful knowledge were found in all the items analysed between the different education groups (*p* = 0.008–<0.001; CI: −2.19 to −0.46; −2.11 to −0.05), except between the secondary vs. BAC and VET for knowledge in wounds (*p* = 0.052; CI: −0.52; 0.00) and between the BAC and VET vs. university in wounds for meaningful knowledge (*p* = 0.200; CI: −0.37; 0.05), in trauma injuries for both knowledge and meaningful knowledge (*p* = 0.466–1.000; CI: −0.41 to −0.16; 0.10 to 0.27), in allergies for both knowledge and meaningful knowledge (*p* = 0.519–1.000; CI: −0.49 to −0.33; 0.13 to 0.18), and in CPR for meaningful knowledge (*p* = 0.050; CI: −0.43; 0.00).

Table 2 shows the differences in knowledge and meaningful knowledge of first aid according to level of education in females. Significant differences were found as a function of academic level for all dimensions and for the total score (*p* = 0.010–<0.001; ES: 0.026–0.370), with a low to moderate effect size.

**Figure 2:**
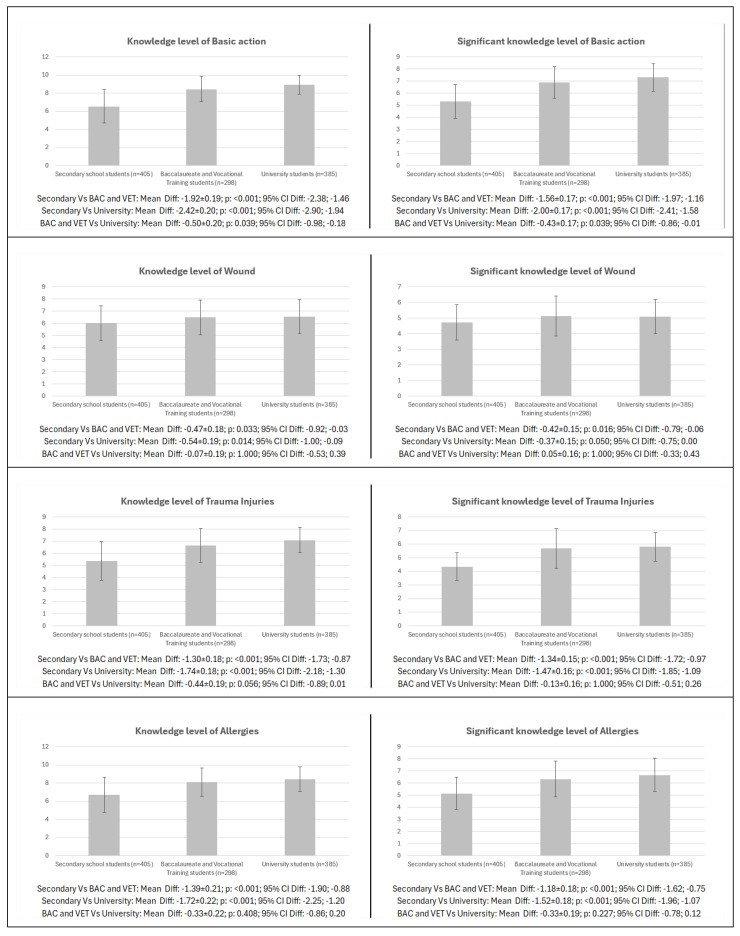

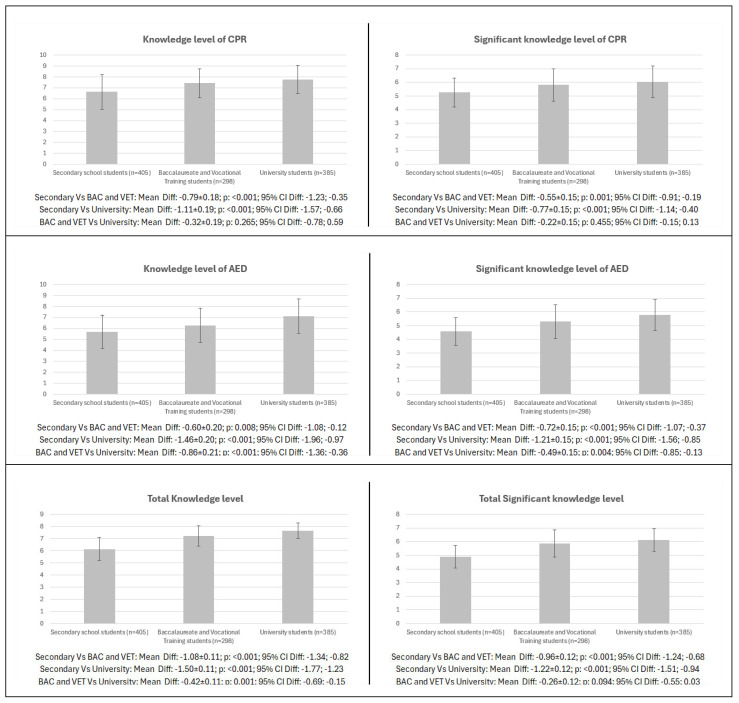
Pairwise differences in first aid knowledge and meaningful knowledge by level of education in females. Legend: CPR: cardiopulmonary resuscitation; AED: automated external defibrillator; BAC: baccalaureate; VET: vocational training.

The pairwise comparison according to level of education for the different dimensions of knowledge and meaningful knowledge level in females is shown in Figure 2. Significant differences were found in knowledge and meaningful knowledge in all items analysed between the different education groups (*p* = 0.039–<0.001; CI: −2.90 to −0.86; −1.94 to −0.01), except between secondary vs. university for meaningful knowledge in wounds (*p* = 0.050; CI: −0.75; 0.00) and except between BAC and VET vs. university for both knowledge and meaningful knowledge in wounds (*p* = 1.000; CI: −0.53 to −0.33; 0.43 to 0.39), trauma injuries (*p* = 0.056–1.000; CI: −0.89 to −0.51; 0.26 to 0.01), allergies (*p* = 0.227–0.408; CI: −0.86 to −0.78; 0.12 to 0.20), and CPR (*p* = 0.265–0.455; CI: −0.78 to −0.15; 0.59 to 0.13) and in meaningful knowledge in the final score (*p* = 0.094; CI: −0.55 to 0.03).

Table 3 shows the differences in knowledge and meaningful knowledge of first aid according to level of education in males. Significant differences were found as a function of academic level for all dimensions and for the total score (*p* < 0.001; ES: 0.029–0.411), with a low to moderate effect size.

**Figure 3:**
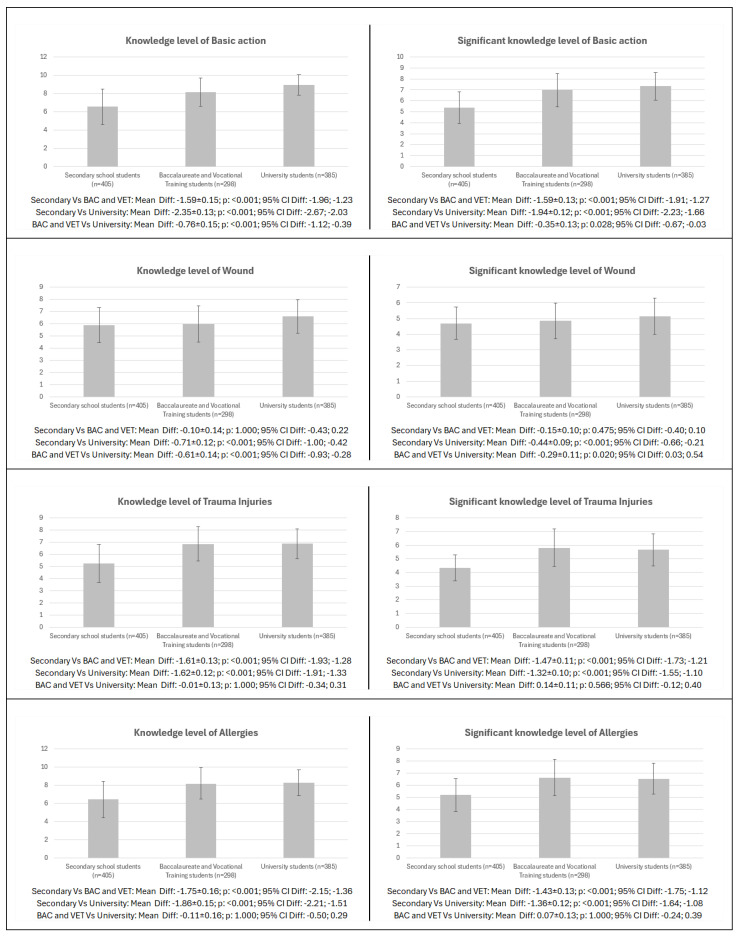

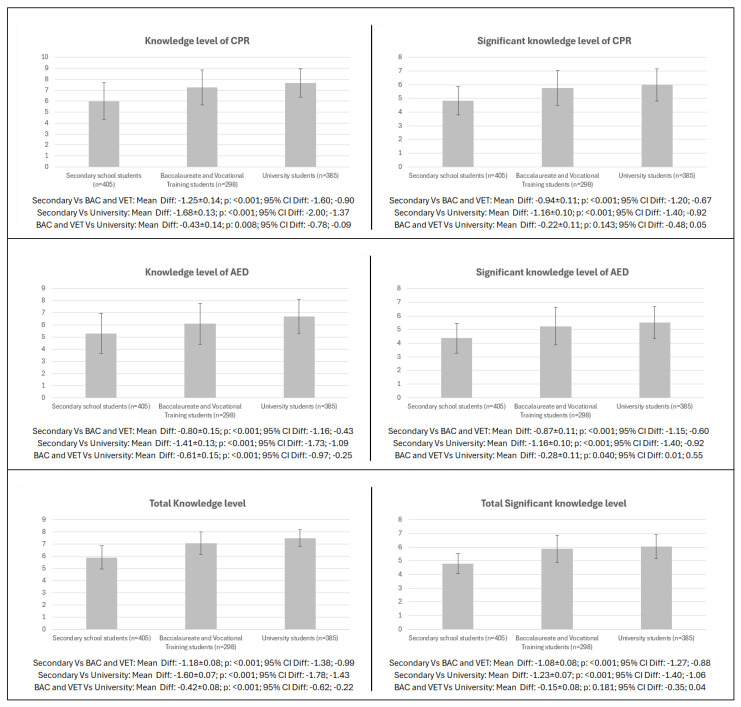
Pairwise differences in first aid knowledge and meaningful knowledge by level of education in males. Legend: CPR: cardiopulmonary resuscitation; AED: automated external defibrillator; BAC: baccalaureate; VET: vocational training.

The pairwise comparison according to level of education for the different dimensions of knowledge and meaningful knowledge level in males is shown in Figure 3. Significant differences in knowledge and meaningful knowledge were found in all items analysed between the different education groups (*p* = 0.039–<0.001; CI: −2.67 to −0.67; −2.03 to −0.03), except between secondary vs. BAC and VET for both knowledge and meaningful knowledge in wounds (*p* = 0.475–1.000; CI: −0.43 to −0.30; 0.22 to 0.10), between BAC and VET vs. university students for both knowledge and meaningful knowledge in trauma injuries (*p* = 0.566–1.000; CI: −0.34 to −0.12; 0.31 to 0.40) and allergies (*p* = 1.000; CI: −0.24 to −0.50; 0.39 to 0.29), and between BAC and VET vs. university students for meaningful knowledge in CPR (*p* = 0.143; CI: −0.48;0.05) and final score (*p* = 0.181; CI: −0.35; 0.04).

Table 4 shows the differences in the importance attached to first aid according to level of education. It was found that secondary school students were more likely to have a low or moderately low interest and knowledge of first aid (*p* < 0.001) and considered this knowledge to be of low or moderately low importance for their personal and professional development (*p* < 0.001), showing significant differences with the other groups.

In contrast, university students rated the interest on this content as high, as well as its importance for both their personal and professional development (*p* < 0.001). However, university students considered that they had a moderately low knowledge of first aid, being surpassed in this item by BAC and VET students (*p* < 0.001).

## 4. Discussion

The present study aimed to analyse the differences in knowledge and meaningful knowledge of first aid according to educational attainment and the influence of gender on these outcomes.

### 4.1. Global Score in First Aid

First aid is pre-hospital care that guarantees better results in future medical care, as it is used to try to keep the victim in the best possible condition while medical care arrives [44]. Specifically, within the sporting context, after an injury, care prior to hospital care is of great importance to reduce pain and suffering [45].

In the present investigation, it was found that significant differences were found in both the knowledge and meaningful knowledge with respect to the overall score on first aid among all stages of education for both the overall sample and for males and females. In this line, university students showed significantly higher scores, followed by BAC and VET students, and lastly by secondary students. The results are consistent with those found in previous studies that indicated that higher educational attainment was found to be a factor associated with better knowledge of first aid [46]. In this line, the university population tends to demonstrate a moderate to good proficiency of first aid, but secondary school students only reach a minimum level of knowledge in this area [47]. This could be because first aid content is included throughout the different stages of education. In this way, secondary school pupils attain only a superficial knowledge, whereas students achieve meaningful learning only in the higher education system, when basic first aid content has been taught on several occasions [48,49]. This is supported by the Spanish educational legislation, which progressively incorporates first aid content from secondary education (Organic Law 3/2020; Decree 235/2022), through baccalaureate (Royal Decree 243/2022), vocational training (Order EDU/90/2022; Royal Decree 570/2023), and into higher education (Resolution of 18 September 2018), thereby allowing students to achieve a priori meaningful learning about basic first aid techniques by the end of their educational stage [22,50,51,52].

However, students in general showed a low level of first aid knowledge, especially when assessing meaningful knowledge. In fact, when analysing the meaningful knowledge dimension, the scores were lower for all groups, showing a lack of confidence by the respondents and/or the internalisation of inadequate protocols. More specifically, despite the higher level among university students, the data found indicates a level of meaningful knowledge that is not adequate for a first aid intervention, according to previous studies [19,23,24]. This highlights the importance of using meaningful knowledge as a measurement, rather than relying solely on simple or theoretical knowledge. In first aid, inaccurate actions, such as improper CPR or wound management, may worsen the condition of the injured person. Therefore, ensuring that knowledge is retained is critical for an effective and safe intervention [53].

Therefore, given the above results, it is important to implement programmes and policies regarding the theoretical and practical teaching of first aid from an early age, to achieve meaningful learning in this subject, where an early and appropriate response can make a significant difference to the death rate and long-term effects of accidents [54]. The inclusion of first aid training across different educational stages would likely help students increase their first aid knowledge as they progress through their academic careers [55]. In this line, previous studies have pointed to the importance of first aid knowledge in effectively performing first aid. Specifically, first aid instruction by teachers, as facilitators of information, is considered an optimal strategy for student satisfaction and knowledge acquisition [56].

### 4.2. Basic Action

In Spain, there are more than 100,000 traffic accidents per year, in addition to more than 5000 other types of accidents [57]. In this context, knowing the basic protocols to follow in the event of an accident is vital [58].

In relation to the basic performance contents, significant differences were found in both the knowledge and meaningful knowledge parameters among all stages of education levels for the overall sample, as well as for men and women separately. University students showed an adequate knowledge in basic actions, followed closely by BAC and VET students, while the scores shown by secondary school students in both the knowledge and meaningful knowledge dimensions were considerably lower. The differences found are due to the fact that, according to Spanish legislation, while first aid content is covered only superficially in the subject of physical education at the secondary school level [52], where the protocols of ‘Protect, Alert, and Rescue’ and the 112 protocol are explained in the first year of CSE, although this content is not included in any of the following CSE courses [15], there is a more in-depth study of these contents in the BAC and VET stages.

More specifically in the baccalaureate, the need to delve into the contents of first aid is indicated, specifically in contents of basic actions such as calling emergency services, while the VET students who are enrolled in health-related or physical-sport disciplines [16] have modules on primary and secondary care included in their curricula [52,59]. The same is true for university students, for whom the European Higher Education Area includes specialised first aid content in degree programmes related to health, exercise, and sports science, based on competences related to health, safety, and emergency intervention [19,59]. Thus, knowledge of basic actions increases with the level of education, with the observation that a greater depth and duration of these contents influences better learning.

### 4.3. Wounds

Wounds are one of the most common accidents that occur on a daily basis [60]. Knowing how to act before such accidents can prevent complications and also avoid overloading the healthcare system [61]. In addition, there are certain complications in the approach to this topic related to the fact that variations in the diagnosis and classification of wounds affect their treatment, and it is necessary to have sufficient knowledge to know what action to take [62]. In this sense, it is worth noting that the wound dimension was the dimension in which the average participant showed the lowest scores. This may be due to factors such as the great variety of factors that must be taken into account when tending to them, in addition to the little importance that is usually given to them, which makes it difficult to understand and learn them correctly [63]. Also, as they are a priori less serious accidents, students may not attach as much importance to them as they do to other types of accidents [64].

After analysing the differences between groups, it was observed that university students showed overall significantly higher values than secondary students for both knowledge and meaningful knowledge. The block on injuries is briefly mentioned in CSE, but it is not until post-compulsory studies that it is explored in more depth [25]. This could explain why CSE students show a lack of knowledge on this topic [65].

On the differences between secondary vs. BAC and VET students, only in the case of females was it found that BAC and VET students were more knowledgeable than secondary school students, with no such differences in males. This may be due to established socio-cultural barriers, resulting in cultural stereotypes, in which females play a more caring role [66]. With wounds being such a common accident and given the low burden of learning this content in the current CSE curriculum, it is possible that some of the learning about how to react to wounds is learned within the household, with females showing a greater interest in this type of knowledge [67]. In accord with this, women tend to show a greater interest in the healthcare field, enrolling in related degree programmes and engaging with topics related to caregiving and health [68,69].

On the contrary, men were the only ones in which university students were found to show a higher knowledge than BAC and VET students. It is possible that men, not being traditionally related to caregiving, may be specifically trained in these contents when they enter specific training courses in the area of sports, where, given the high incidence of injuries occurring during sports practice [70], these topics are specifically addressed [71].

### 4.4. Trauma

Traumas are injuries that are generally associated with concussions, which occur as a result of traffic accidents, falls, and precipitation [72]. These are highly serious accidents, which lead to a high percentage of admissions to Intensive Care Units, and which have a large number of complications that are aggravated if the first aid action is not adequate [72]. Therefore, dealing with trauma cases is highly relevant due to the high frequency of such accidents and the seriousness they entail [73,74]. In this dimension, university students were found to have similar knowledge and meaningful knowledge as BAC and VET students, with significant differences in both with respect to secondary students, regardless of gender. This could be due to the fact that trauma is dealt with in a very general manner in secondary schools, which means that this population has practically no knowledge of the subject [75]. It is not until post-compulsory training that the initial assessment of the victim is included, covering fractures, sprains, dislocations, contusions, and polytraumatisms [25]. The acquisition of basic knowledge in this area in BAC and VET could explain why there are no differences with respect to university students [25]. Not surprisingly, previous studies have shown that a large proportion of university students do not provide first aid for this type of accident due to a lack of knowledge or nervousness [47], which could demonstrate the need for specific training in trauma management and decision making, up to the final stages of training [76].

### 4.5. Allergy

Cases of food allergy, insect stings, or medication allergy have increased considerably over the last few years, mainly affecting adults [77]. In this dimension, it was found that university students had similar knowledge and meaningful knowledge to BAC and VET students, with significant differences in both with respect to secondary school students, regardless of gender. This could be due to the fact that the content of allergy first aid is not even included in the CSE curriculum in the Region of Murcia [15]. Therefore, it is not until post-compulsory training that students are taught some content related to this block [22,52,59], although it is true that it is rarely taught in great depth, which could explain the lack of an increase in knowledge between the different post-compulsory education stages [25]. In the same vein, previous studies have pointed out that, even when post-compulsory education has been achieved, knowledge about allergies is low [78]. For all of the above reasons, it is necessary to expand training in these contents throughout the different stages of education, with specific training on allergies and the main ways to act when dealing with these emergencies.

### 4.6. Cardiopulmonary Resuscitation

The high frequency of heart attacks as emergencies in our society justifies the importance of knowledge of basic first aid actions for the first response to an accident [79]. In Spain, one in three people currently die of cardiovascular disease, with acute myocardial infarction being the main cause of death [80]. In such situations, proper CPR is essential to save lives as part of the first aid protocol, through chest compressions in case of cardiac arrest [81]. In this dimension, it was found that, in general, university students had greater knowledge and meaningful knowledge than BAC and VET students, with significant differences in both with respect to secondary school students, regardless of gender. CPR is addressed as content in the 4th year of CSE, so it is not until this last year of compulsory education that students begin to become familiar with this technique [15,25]. As a result, secondary school students lack confidence in identifying and responding to a cardiac arrest [55]. Meanwhile, bachelor students learn how to identify a cardiac arrest and start CPR, so this specific learning could explain the increase in knowledge on this topic [25,52,82]. In VET, CPR is visualised within the basic life support techniques, where it is presented in specific modules in the areas of health and sport, both theoretically and practically [59,83]. Finally, at university, first aid training changes according to the degree and university, being more present in students in the areas of health and sport, with CPR forming part of the specific compulsory training [22,47].

In addition to the above, the assessment of the lines of action in the event of needing to carry out CPR has been assessed in a theoretical manner. In this sense, previous research has shown that theoretical training on CPR does not translate into real practical mastery without specific courses for this purpose [84]. Therefore, future research should analyse the differences in students’ practical responses to such circumstances according to level of education and also analyse the need to offer specific CPR training courses with a combination of theoretical and practical aspects [85], given that this type of training is a key requirement for developing competency in resuscitation skills, an area where students have shown a strong interest, which does not manifest itself in a greater practical mastery of CPR [86].

### 4.7. Automated External Defibrillator

Defibrillators are fundamental for the rapid recovery of the affected person, given their effectiveness as compared to manual methods, and knowledge on their use is currently considered to be basic in the awareness of correct first aid protocols [87]. In relation to the AED content, significant differences were found in both the knowledge and meaningful knowledge parameters between all levels of education for both the general sample and for females and males. University students showed a higher knowledge in both the knowledge and meaningful knowledge dimensions in this content block, followed by BAC and VET students, and secondary school students, respectively. The use of AEDs is not addressed as content until the 4th year of CSE, so it is not until this last year of compulsory education that students start to become familiarised with the use of this element [15]. As a consequence, secondary school students are unaware of the actual use of an AED, having difficulties in recognising its functions [88]. A higher level of education results in greater skills in the use of AEDs due to a greater interest in participating in AED training, because of an understanding of the need for proper resolution of emergencies, but also due to the increased training on this content received in the educational sphere [89].

In this respect, in BAC, it is specified that training on the use of the defibrillator has to be provided [52], while, in VET, it is present in specific titles as part of basic life support techniques, aligning with the needs of the current market [59]. Finally, at university level, there is no specific mention of the use of AEDs, but content related to health and safety in sport is mentioned, promoting a greater awareness of risk prevention [22,90]. As a consequence of the above, it is important to incorporate basic life-saving procedures, as well as CPR and AED training, into educational curricula and public health programmes to improve bystander response to syncope and out-of-hospital cardiac arrest [91]. Such programmes are necessary because it was found that, although students are willing to learn these techniques, training programmes in this area are generally insufficient [92].

### 4.8. Gender Differences

Gender differences were significant in the CPR and AED dimensions. Previous studies have already suggested that females may be more knowledgeable about first aid topics [93]. The gender differences found in these dimensions could be due to the fact that females may show a greater engagement or confidence with these topics, as they consider them more relevant to first aid, possibly due to cultural factors or a greater participation in healthcare-oriented learning environments [94]. Another possible explanation could be that females tend to have a greater vocation for healthcare careers [95]. It is possible, therefore, that from pre-university stages they are interested in learning contents related to their possible future training, especially those that they may consider more relevant [96]. These issues need to be tested in future research.

However, there were no gender differences in the other dimensions. This could be because other first aid topics may be considered less directly related to the survival of the injured person and therefore may have been given less specific weight within the informal learning of first aid in the home [97]. This type of learning is more associated with women, given the stereotypes of women caring for others.

As a consequence of the above, the first hypothesis can be partially accepted, as it was found that the level of knowledge in first aid should be higher and more consistent as the student acquires higher levels of education, regardless of gender in most cases, although there were dimensions in which no differences in knowledge were found between BAC and VET students and university students. Similarly, gender appeared to have a significant influence on the differences between levels of education in some dimensions, such as wounds.

### 4.9. Importance Given to First Aid Knowledge

The second objective of the present research was to determine the differences in the importance given to first aid according to level of education. It was found that interest in first aid increased with the level of education, as did the perception of its importance for personal and professional development. These results are in line with previous studies that found that interest in and perceived importance of first aid progress with the level of education, with a greater willingness to receive training in this area, with more confidence in its performance, but with limitations in practical knowledge [98]. This could be due to the fact that first aid assessment can be increased by several factors such as cognitive and emotional maturity or the professional relevance of the skills learned, especially in the case of health- and sports-related training [99,100,101,102]. It may also be due to having witnessed accidents or received specific training previously, which would lead to a greater awareness of the need for training in this area [102,103].

In addition, it was found that, in general, the knowledge reported was low at all levels. This is in line with previous research, where it was found that students do not feel that they have sufficient knowledge, but factors such as being female, receiving training, and participating in educational training on first aid are found to improve the perception of first aid knowledge [104]. This discrepancy between interest and self-perceived competence indicates a gap that must be addressed through more comprehensive, practical, and confidence-building training. Previous studies reinforce the idea that, although students place importance on first aid at all stages of education, they have poor knowledge and need better educational intervention [102,105].

Given the above, the second hypothesis is accepted, as it was found that students in higher education place more importance on first aid knowledge than students in lower levels of education.

### 4.10. Limitations of This Study

The present research is not without limitations. The choice of a convenience sample limits the generalisability of the results to other populations, especially towards VET students and students of university degrees in other fields of knowledge. In this line, the fact that the vocational training and university degree students belonged to the field of sports and had first aid training included in their academic programme may limit the generalisability of the results to students from other academic disciplines. Thus, it is not possible to generalise the findings to students enrolled in other vocational or university programmes, as the inclusion and depth of first aid training varies significantly across curricula, depending on the specific focus of the degree. For example, it would be interesting to replicate this study with other types of students who do not receive any first aid training, such as humanities or engineering students, in order to compare first aid knowledge between VET and university students according to their field of study, especially since no individual is exempt from encountering accidents in everyday life and needs to know how to act.

Also, the main limitation was the lack of a sufficiently large sample of BAC and VET students to allow for separate group analyses; in the present study, they were analysed jointly following the methodology of previous studies [41]. Therefore, both groups were combined, as they represent educational pathways undertaken after completing compulsory education. These two groups are situated in an educational stage that follows compulsory schooling but precedes university-level studies. However, future studies should explore whether there are also differences between BAC and VET students in this domain.

Finally, previous studies have shown that parents’ and caregivers’ knowledge of first aid could influence young people’s knowledge of first aid [106], so it would be advisable to take into account the parents’ knowledge in future studies in order to analyse the specific weight that this specific context or formal education has on the students’ knowledge.

### 4.11. Practical Implications and Recommendations

Based on the results obtained, it is evident that, while knowledge of first aid improves progressively with the level of education, students generally show a limited mastery of meaningful and practical knowledge across all dimensions assessed. The results of this study underscore the need to ensure equitable and comprehensive first aid education across all academic levels and disciplines and to consider tailored approaches that address gender disparities in specific skill areas.

Early first aid education can have a profound long-term impact on society by fostering a more resilient, responsible, and health-conscious population. When individuals are trained from a young age to respond effectively in emergency situations, the likelihood of timely and appropriate intervention increases, potentially reducing mortality and the severity of injuries in everyday accidents, natural disasters, or public emergencies. Over time, this contributes to a culture of preparedness and civic responsibility, where helping others becomes a social norm rather than an exception. Additionally, widespread early education in first aid can ease the burden on emergency medical services by enabling bystanders to provide immediate care before the emergency professionals arrive, thus improving overall public health outcomes. In the long run, such education supports not only safer schools and communities but also strengthens societal cohesion by empowering citizens with life-saving skills that benefit everyone.

Although the current educational legislation provides a framework for introducing first aid throughout the academic journey, the findings suggest that the depth and continuity of this content remain insufficient to guarantee effective actions in real-life emergencies. Thus, the information provided in this article may be of interest to the education sector when designing curricula. More specifically, policymakers should prioritise that the laws provide a context that allows achieving a comprehensive approach to first aid education that blends practical experience with structured learning. Introducing simulated emergency scenarios into the curriculum can significantly enhance the students’ ability to respond effectively under pressure, especially when adapted to be age-appropriate and context-specific. Making first aid workshops mandatory—ideally on an annual basis—ensures that students retain and update their knowledge over time, rather than receiving one-time instruction that quickly fades. Additionally, offering extracurricular certifications through partnerships with health organisations provides a formal recognition of skills and motivates student engagement, particularly if linked to academic credit or community service.

This highlights the urgent need to reinforce the teaching of first aid not only through theoretical content but through meaningful, experiential, and competence-based learning from early educational stages. In this line, it may be useful for teachers in various disciplines, such as biology or physical education, to address first aid from a multidisciplinary perspective. In addition, active methodologies such as game-based learning have been shown to foster a positive learning environment and enhance students’ confidence in applying first aid techniques [107]. Therefore, the recommendation is made to implement comprehensive first aid programmes that span all levels of education, integrating both theoretical understanding and practical application, with particular emphasis on critical areas such as CPR, trauma, allergy response, and AED use. Training should also account for gender-based engagement and address cultural or societal biases that may influence the learning outcomes.

However, despite the central role of teachers in the transmission of knowledge, some studies suggest that they often lack adequate training in this area [40,108]. Teachers play a key role in shaping students’ first aid competencies, so they should be prepared and supported as key facilitators in this process, given their pivotal role in promoting first aid education. Promoting student confidence, reinforcing practical training, and ensuring curricular coherence are essential for bridging the gap between the importance attributed to first aid by the students and their actual competence to intervene effectively in emergencies. Therefore, teachers should receive certified training in first aid themselves, ideally through accredited programmes, to ensure they have both the knowledge and confidence to teach the content accurately and safely. Integrating first aid instruction into relevant subjects—such as physical education or biology—can make learning more contextual and meaningful. Teachers should incorporate active learning strategies, such as role-playing, peer teaching, and problem-solving around simulated emergency scenarios, to promote the development of meaningful, not just theoretical, knowledge. Whenever possible, they should collaborate with external professionals to enrich lessons with real-world expertise. Finally, teachers should assess students not only through written tests but also via practical demonstrations, encouraging both skill mastery and the confidence needed to act in real emergencies. By integrating these strategies and ensuring that educators are properly trained to deliver first aid content, institutions can cultivate a culture of preparedness and responsibility across all educational stages.

Therefore, training students at all levels of education in first aid is essential for building a society that is prepared to adequately respond with the skills necessary to assist the injured [4,107]. Moreover, extending this type of research to students in non-health-related degrees could provide a broader understanding of training needs and further inform educational policy and curriculum design.

## 5. Conclusions

This study confirms that level of education is a determining factor in first aid knowledge, with university students showing the highest and most consistent levels of understanding in this area. They are followed by BAC and VET students, while secondary school students appeared to be the least prepared group. The results underline that perceived confidence is not necessarily related to actual first aid competence. For this reason, it is essential to not only transmit knowledge but also to foster self-awareness and confidence when facing emergency situations.

Some areas, such as CPR and wound care, showed a clear progression as students advance through the education system. The inclusion of mandatory practical workshops that integrate emergency simulations is recommended to improve confidence and skills in real situations. In addition, the application of active methodologies, such as game-based learning, can enhance knowledge retention [109]. Promoting recognised extracurricular certifications in first aid could encourage student participation and commitment. Finally, it is crucial to train teachers in these competencies to ensure effective and cross-cutting teaching in the curriculum [110]. These actions will help strengthen students’ preparedness to intervene appropriately in emergencies, reducing risks and improving public health.

In contrast, others—such as trauma and allergic reactions—did not show a significant improvement, suggesting possible gaps in how these topics are addressed in the curriculum. Gender did not appear to be a relevant factor overall, except in specific areas such as wound care. This suggests that academic training may have a greater impact on first aid knowledge than gender. This approach would help foster a greater awareness, preparedness, and responsiveness in emergency situations. Future research should explore differences within the BAC and VET student group, given the recent diversification of these academic pathways. In addition, it is essential that teachers receive adequate training in first aid and adopt active, innovative methodologies that enhance meaningful and effective learning of these skills.

## Figures and Tables

**Table 1:** Differences in knowledge and meaningful knowledge of first aid according to level of education and influence of gender.

Score	Groups			ANOVA			Gender Covariate		
	Secondary School Students (*n* = 405)	Baccalaureate and Vocational Training Students (*n* = 298)	University Students (*n* = 385)	F	*p*	ES (η^2^)	F	*p*	ES (η^2^)
Knowledge level									
Basic action	6.56 ± 1.92	8.28 ± 1.50	8.93 ± 1.10	243.87	<0.001	0.310	0.56	0.452	0.001
Wounds	5.93 ± 1.44	6.19 ± 1.49	6.59 ± 1.37	21.26	<0.001	0.038	3.49	0.062	0.003
Trauma Injuries	5.29 ± 1.58	6.78 ± 1.41	6.94 ± 1.20	161.59	<0.001	0.230	0.23	0.630	<0.001
Allergy	6.51 ± 1.98	8.15 ± 1.66	8.33 ± 1.39	132.98	<0.001	0.197	0.95	0.329	0.001
CPR	6.18 ± 1.67	7.31 ± 1.49	7.69 ± 1.30	107.49	<0.001	0.165	10.54	0.001	0.010
AED	5.42 ± 1.64	6.17 ± 1.65	6.83 ± 1.45	79.30	<0.001	0.128	11.24	0.001	0.010
Total	5.98 ± 0.96	7.15 ± 0.90	7.55 ± 0.69	356.87	<0.001	0.397	10.18	0.001	0.009
Meaningful knowledge level									
Basic action	5.37 ± 1.43	6.94 ± 1.45	7.33 ± 1.23	224.53	<0.001	0.293	0.53	0.466	<0.001
Wounds	4.71 ± 1.06	4.96 ± 1.20	5.12 ± 1.14	13.81	<0.001	0.025	1.33	0.249	0.001
Trauma Injuries	4.35 ± 0.96	5.77 ± 1.40	5.71 ± 1.14	182.81	<0.001	0.252	0.03	0.871	<0.001
Allergy	5.18 ± 1.35	6.51 ± 1.51	6.58 ± 1.31	125.59	<0.001	0.188	0.49	0.485	<0.001
CPR	4.96 ± 1.08	5.79 ± 1.24	6.00 ± 1.17	87.92	<0.001	0.139	5.98	0.015	0.005
AED	4.44 ± 1.07	5.27 ± 1.33	5.60 ± 1.14	103.04	<0.001	0.160	5.18	0.023	0.005
Total	4.83 ± 0.77	5.87 ± 0.99	6.06 ± 0.86	225.73	<0.001	0.294	0.93	0.335	0.001

CPR: cardiopulmonary resuscitation; AED: automated external defibrillator.

**Table 2:** Differences in knowledge and meaningful knowledge of first aid according to level of education in females.

Score	Groups			ANOVA		
	Secondary School Students (*n* = 122)	Baccalaureate and Vocational Training Students (*n* = 117)	University Students (*n* = 106)	F	*p*	ES (η^2^)
Knowledge level						
Basic action	6.53 ± 1.85	8.45 ± 1.42	8.95 ± 1.04	85.64	<0.001	0.334
Wounds	6.02 ± 1.44	6.49 ± 1.45	6.56 ± 1.40	5.00	0.007	0.028
Trauma Injuries	5.36 ± 1.59	6.66 ± 1.42	7.10 ± 1.06	49.36	<0.001	0.224
Allergy	6.70 ± 1.94	8.09 ± 1.55	8.42 ± 1.36	35.97	<0.001	0.174
CPR	6.63 ± 1.60	7.42 ± 1.31	7.75 ± 1.30	19.06	<0.001	0.100
AED	5.69 ± 1.51	6.29 ± 1.57	7.15 ± 1.56	25.46	<0.001	0.130
Total	6.15 ± 0.95	7.23 ± 0.84	7.65 ± 0.66	100.40	<0.001	0.370
Meaningful knowledge level						
Basic action	5.31 ± 1.40	6.88 ± 1.32	7.31 ± 1.15	75.82	<0.001	0.307
Wounds	4.72 ± 1.13	5.14 ± 1.27	5.09 ± 1.09	4.65	0.010	0.026
Trauma Injuries	4.35 ± 1.02	5.70 ± 1.45	5.82 ± 1.06	54.67	<0.001	0.242
Allergy	5.15 ± 1.32	6.34 ± 1.49	6.67 ± 1.37	37.98	<0.001	0.182
CPR	5.26 ± 1.07	5.81 ± 1.20	6.04 ± 1.18	13.85	<0.001	0.075
AED	4.58 ± 1.01	5.30 ± 1.24	5.79 ± 1.11	33.32	<0.001	0.163
Total	4.90 ± 0.83	5.86 ± 1.00	6.12 ± 0.83	60.47	<0.001	0.261

CPR: cardiopulmonary resuscitation; AED: automated external defibrillator.

**Table 3:** Differences in knowledge and meaningful knowledge of first aid according to level of education in males.

Score	Groups			ANOVA		
	Secondary School Students (*n* = 283)	Baccalaureate and Vocational Training Students (*n* = 181)	University Students (*n* = 279)	F	*p*	ES (η^2^)
Knowledge level						
Basic action	6.57 ± 1.95	8.17 ± 1.54	8.93 ± 1.12	159.94	<0.001	0.302
Wound	5.90 ± 1.44	6.00 ± 1.48	6.61 ± 1.37	19.51	<0.001	0.050
Trauma Injuries	5.26 ± 1.57	6.87 ± 1.40	6.88 ± 1.24	113.85	<0.001	0.235
Allergy	6.43 ± 1.99	8.19 ± 1.73	8.29 ± 1.41	96.96	<0.001	0.208
CPR	5.99 ± 1.67	7.24 ± 1.60	7.67 ± 1.30	90.84	<0.001	0.197
AED	5.30 ± 1.68	6.10 ± 1.70	6.71 ± 1.39	55.84	<0.001	0.131
Total	5.91 ± 0.96	7.09 ± 0.93	7.51 ± 0.69	258.47	<0.001	0.411
Meaningful knowledge level						
Basic action	5.39 ± 1.45	6.98 ± 1.53	7.33 ± 1.26	149.10	<0.001	0.287
Wound	4.70 ± 1.03	4.85 ± 1.14	5.14 ± 1.15	11.15	<0.001	0.029
Trauma Injuries	4.35 ± 0.94	5.82 ± 1.36	5.67 ± 1.17	129.05	<0.001	0.259
Allergy	5.19 ± 1.37	6.63 ± 1.51	6.55 ± 1.28	89.59	<0.001	0.195
CPR	4.83 ± 1.05	5.77 ± 1.27	5.99 ± 1.17	77.48	<0.001	0.173
AED	4.37 ± 1.08	5.25 ± 1.39	5.53 ± 1.15	70.75	<0.001	0.161
Total	4.81 ± 0.74	5.88 ± 0.99	6.04 ± 0.87	166.14	<0.001	0.310

CPR: cardiopulmonary resuscitation; AED: automated external defibrillator.

**Table 4:** Differences in the interest, importance given, and self-perception of knowledge about first aid according to level of education.

Descriptors	Secondary School Students (*n* = 405) (*n*, % and Adj. Res.)	Baccalaureate and Vocational Training Students (*n* = 298) (*n*, % and Adj. Res.)	University Students (*n* = 385) (*n*, % and Adj. Res.)	χ^2^	*p*	Cramer’s V
Interest you have in first aid	Low: 72 (17.8%). Adj.: 9.0 Moderate Low: 158 (39.0%). Adj.: 10.5 Moderate High: 143 (35.3%). Adj.: −2.9 High: 32 (7.9%). Adj.: −11.8	Low: 12 (4.0%). Adj.: −3.0 Moderate Low: 47 (15.8%). Adj.: −3.0 Moderate High: 145 (48.7%). Adj.: 3.2 High: 94 (31.5%). Adj.: 1.2	Low: 4 (1.0%). Adj.: −6.3 Moderate Low: 34 (8.8%). Adj.: −7.7 Moderate High: 158 (41.0%). Adj.: 0.0 High: 189 (49.1%). Adj.: 10.8	289.83	<0.001	0.36
Importance of first aid for your personal development	Low: 60 (14.8%). Adj.: 7.9 Moderate Low: 127 (31.4%). Adj.: 10.8 Moderate High: 156 (38.5%). Adj.: 0.3 High: 62 (15.3%). Adj.: −12.5	Low: 13 (4.4%). Adj.: −2.0 Moderate Low: 32 (10.7%). Adj.: −2.8 Moderate High: 121 (40.6%). Adj.: 1.1 High: 132 (44.3%). Adj.: 2.0	Low: 2 (0.5%). Adj.: −6.1 Moderate Low: 13 (3.4%). Adj.: −8.3 Moderate High: 135 (35.1%). Adj.: −1.4 High: 235 (61.0%). Adj.: 10.8	275.33	<0.001	0.36
Importance of first aid for your profession	Low: 118 (29.1%). Adj.: 11.0 Moderate Low: 156 (38.5%). Adj.: 11.3 Moderate High: 84 (20.7%). Adj.: −4.1 High: 47 (11.6%). Adj.: −13.5	Low: 30 (10.1%). Adj.: −2.3 Moderate Low: 42 (14.1%). Adj.: −3.3 Moderate High: 106 (35.6%). Adj.: 3.4 High: 120 (40.3%). Adj.: 1.2	Low: 5 (1.3%). Adj.: −9.0 Moderate Low: 26 (6.8%). Adj.: −8.4 Moderate High: 115 (29.9%). Adj.: 1.0 High: 239 (62.1%). Adj.: 12.5	368.45	<0.001	0.41
Level of knowledge you consider you currently have about first aid	Low: 153 (37.8%). Adj.: 5.9 Moderate Low: 179 (44.2%). Adj.: −2.4 Moderate High: 54 (13.3%). Adj.: −3.1 High: 19 (4.7%). Adj.: −0.8	Low: 48 (16.1%). Adj.: −5.2 Moderate Low: 149 (50.0%). Adj.: 0.4 Moderate High: 78 (26.2%). Adj.: 4.2 High: 23 (7.7%). Adj.: 2.1	Low: 98 (25.5%). Adj.: −1.1 Moderate Low: 205 (53.2%). Adj.: 2.1 Moderate High: 65 (16.9%). Adj.: −0.8 High: 17 (4.4%). Adj.: −1.1	53.75	<0.001	0.16

## Data Availability

The database can be obtained from the corresponding author upon reasonable request.

## Supplementary Materials

The following supporting information can be downloaded at: https://www.mdpi.com/article/10.3390/healthcare13131507/s1, File S1: Supplementary document 1: First aid knowledge level assessment questionnaire.

## Abbreviations

The following abbreviations are used in this manuscript:

AED	Automated External Defibrillator
BAC	Baccalaureate
CPR	Cardiopulmonary Resuscitation
SD	Standard Deviation
V	Cramer’s V test
VET	Vocational Education and Training
