# Supplementary material for: Assessment of First Aid Knowledge at Different Stages of Education
Source: Healthcare (Basel). 2025 Jun 24;13(13):1507. doi: 10.3390/healthcare13131507 (PMC12249045; doi:10.3390/healthcare13131507)
Supplement: Supplementary file 1 [file healthcare-13-01507-s001.zip › healthcare-3607529-supplementary.pdf]

SUPPLEMENTARY DOCUMENT 1: FIRST AID KNOWLEDGE LEVEL ASSESSMENT QUESTIONNAIRE

The purpose of this questionnaire is to discover how much First Aid training you have. You should select only one answer per item. Try to answer as truthfully as possible, and choose the option that best suits your possible action.

NOTE: Completion of this questionnaire implies that I have been informed of the study and research procedures of the Project entitled "Assessment of knowledge and performance in lifesaving and lifeguarding in the Spanish population" and of the research procedures. Completion of this questionnaire will be considered informed consent to participate in this research. The data collected will be treated in compliance with Regulation (EU) 2016/679 of the European Parliament and of the Council, of 27 April 2016, Royal Decree-Law 5/2018, of 27 July, and Organic Law 15/1999, of 13 December, on the Protection of Personal Data.

1. My sex/gender is:
   a) Male
   b) Female
2. Age: ………. years old.
3. What are you currently studying?
   a) Compulsory Secondary Education. Specify academic year: ……
   b) Baccalaureate. Specify academic year: ……
   c) Vocational Education and Training. Specify the name of the training: ……………………………………………………………………………………….
   d) University Degree. Specify the name of the training: ……………………………………………………………………………………….

Rate the following items in relation to the provided scale

4. INTEREST you have in First Aid content
   a) Little   b) Some   c) Quite a lot   d) A lot

5. IMPORTANCE of this subject for your personal training

   a) Little b) Some c) Quite a lot d) A lot

6. IMPORTANCE of this subject for your profession

   a) Little b) Some c) Quite a lot d) A lot

7. LEVEL of knowledge you feel you currently possess

   a) Little b) Some c) Quite a lot d) A lot

TEST/ASSESSMENT of First Aid knowledge

The questionnaire aims to determine the level of knowledge in First Aid. It presents scenarios and/or questions in which there are no universal true answers, so you must indicate the answer that most closely matches the action you would take in the scenario presented. In addition, for each question, you must indicate the degree of certainty of your answer.

Below, you will find a series of questions about how you would deal with common accidents in everyday life. You should select only one answer per item. Try to answer as truthfully as possible, and choose the option that best suits your possible action.

8. You are on the road, and you come across an accident. Part of the vehicle occupies the road. What do you think would be the most appropriate course of action?

   a) The first thing to do is to assist the accident victims, then to alert the emergency services, and, finally, to protect the scene so that there are no more accidents.

   b) I do not have to do anything, because I am not an expert in the field.

   c) The first thing to do is to protect the site of the accident, then to rescue the accident victims, and, finally, to alert the emergency services when I know what is happening to the accident victims.

   d) The first thing to do is to protect the scene, then to alert the emergency services, and, finally, to rescue to the accident victims.

   How confident are you in your answer to this question?

a) Very uncertain. b) Somewhat certain. c) Certain. d) Very certain.

9. In an external bleeding on the forearm situation, which is actively bleeding, if direct pressure is applied to the wound for more than 5 minutes and the bleeding does not stop…

   a) Apply a tourniquet to the forearm above the bleeding area.

   b) Continue direct pressure, no need to add or remove gauze.

   c) Continue direct pressure of the wound, placing new gauze on top of old gauze pads.

   d) Continue direct pressure of the wound by elevating the affected limb, removing the soaked gauze, and placing new gauze pads.

   How confident are you in your answer to this question?

   a) Very uncertain. b) Somewhat certain. c) Certain. d) Very certain.

10. During CPR of an adult, rescue breathing will be performed...

    a) Just enough to raise the chest a little higher than normal to ensure that the person is effectively oxygenated.

    b) Giving two breaths of one second each.

    c) Giving two breaths of less than 1 second each.

    d) Before chest compressions are started.

    How confident are you in your answer to this question?

    a) Very uncertain. b) Somewhat certain. c) Certain. d) Very certain.

11. What should you do if you have a glenohumeral (shoulder) dislocation?

    a) Immobilise the affected joint with a sling (90° elbow) and apply cold.

    b) Try to reduce it by putting the joint back in place.

    c) Immobilise the joint with a sling and apply heat.

d) Immobilise the arm extended over the body.

How confident are you in your answer to this question?

a) Very uncertain. b) Somewhat certain. c) Certain. d) Very certain.

12. A woman starts to feel dizzy. She is weak and tries to lean on something. Her face is pale, and her friends comment that this has happened to her before. She has syncope. What would be the most appropriate action to take?

a) I would sit her down and put her head between her legs to raise her blood pressure, and, when she is better, I would stand her up.

b) I would leave her standing and give her water because, as it has happened more than once, it is normal that she will recover.

c) I would lay her on the floor and, when she is better, I would help her to get up.

d) I would lay her on the floor in the shock position (legs elevated), loosen her tight clothes, and, when she is better, sit her up for a while and try to lift her very slowly.

How confident are you in your answer to this question?

a) Very uncertain. b) Somewhat certain. c) Certain. d) Very certain.

13. You are faced with a person who is in cardiac arrest (unconscious and not breathing). When you remove their clothing to apply the AED pads you notice that they have a pacemaker.

a) Do not use the AED, as this would destroy the pacemaker and, if the cardiac arrest is reversed, the patient would return to cardiac arrest.

b) We will place the pads, one on his back and one on his chest, away from the pacemaker.

c) Use the AED, avoiding placing the pads on top of the pacemaker.

d) Follow the steps of the AED by placing the pads under both armpits.

How confident are you in your answer to this question?

a) Very uncertain. b) Somewhat certain. c) Certain. d) Very certain.

14. If a person has a finger amputated, how should we preserve the amputated part?

a) Cover the limb with sterile moistened sterile gauze, place it in a bag, and place the bag in another sealed bag placed in ice slurry.

b) Place the limb in a bag with crushed ice.

c) Wash the limb with alcohol and place it in a bag with ice.

d) Do not handle the amputated part, and call the emergency services.

How confident are you in your answer to this question?

a) Very uncertain. b) Somewhat certain. c) Certain. d) Very certain.

15. If we are faced with an unconscious, non-breathing 9-year-old, who is undergoing CPR, what is the compression-to-ventilation ratio?

a) 30 compressions and 2 ventilations.

b) 30 compressions and 1 ventilation regardless of whether he raises the chest.

c) As it is a child, I would give 5 ventilations and 30 compressions.

d) 5 compressions, 1 ventilation, as long as there was another collaborator.

How confident are you in your answer to this question?

a) Very uncertain. b) Somewhat certain. c) Certain. d) Very certain.

16. We know that a person is diabetic, and we find them conscious (they can eat), with hypoglycaemia symptoms possibly due to their illness (sweating, coldness, muscle weakness, etc.). The action we will take is...

a) Administer a fast-acting carbohydrate, and call the emergency services... * Never give them sugar, as they will be too weak.

b) Never administer sugar, as hyperglycaemia can aggravate the situation.

c) Never give sugar, as low blood sugar can aggravate the situation.

d) Give water, lie on the back with legs elevated 20-30 cm, and call the emergency services.

How confident are you in your answer to this question?

a) Very uncertain. b) Somewhat certain. c) Certain. d) Very certain.

17. You are cooking and you burn yourself with oil on one hand. The area becomes red, and a blister appears. What action would you take?

    a) I would put the hand under the cool running water for at least 10 minutes, protect the burn area until it is assessed by medical services.

    b) I would add toothpaste to the area of the burn and do not puncture the blister.

    c) Initiate treatment with cool compress wrapped in a cotton cloth on the burned area.

    d) I would use hydrogen peroxide and vinegar to disinfect the wound.

    How confident are you in your answer to this question?

    a) Very uncertain. b) Somewhat certain. c) Certain. d) Very certain.

18. In the case of a bruise where a large bruise appears...

    a) A topical antiseptic will be applied to the area and covered with a gauze.

    b) Ice will be applied directly to the affected area.

    c) We will do nothing as it must first be checked by medical personnel.

    d) Localized cold therapy will be applied, protecting it with gauze between the skin and the ice, to reduce bleeding.

19. You find an 8-year-old child in cardiac arrest, and when you open the AED case you notice that there are only adult pads... I only perform basic life support because I do not have paediatric pads.

    a) Turn on and follow AED instructions, applying adult pads to child.

    b) I use a single pad to reduce the shock to be delivered to the child.

    c) Notify emergency services of the situation, begin basic CPR, and wait for paediatric pads to arrive with the emergency services.

    How confident are you in your answer to this question?

    a) Very uncertain. b) Somewhat certain. c) Certain. d) Very certain.

20. Regarding chest compressions in a CPR performed on an adult...

   a) It is important to give chest compressions; the compression depth and rate are not so important.

   b) At least two people must be present to perform them correctly.

   c) 100–120 compressions per minute and the sternum should be compressed at least 5 centimetres.

   d) It is very important to give chest compressions to the patient; if you are not trained, the ventilations are not so important.

   How confident are you in your answer to this question?

   a) Very uncertain. b) Somewhat certain. c) Certain. d) Very certain.

21. When faced with a severe bleeding from the arm, the most appropriate course of action is...

   a) Elevate the arm with the wound without applying direct pressure with gauze.

   b) Apply direct compression to the wound with gauze or a garment.

   c) Apply a haemostatic tourniquet immediately if the person is dizzy and pale.

   d) Apply direct compression to the pressure point artery.

   How confident are you in your answer to this question?

   a) Very uncertain. b) Somewhat certain. c) Certain. d) Very certain.

22. You are responding to cardiac arrest on your own when the emergency services arrive with an AED:

   a) I continue to perform CPR while the emergency services check the AED.

   b) I immediately stop my performance so that they can continue to assist him/her.

   c) They will tell me when I should finish chest compressions, and they will continue with CPR and AED techniques.

   d) I will continue CPR, even if they tell me to stop.

   How confident are you in your answer to this question?

a) Very uncertain. b) Somewhat certain. c) Certain. d) Very certain.

23. You see a person who has twisted their left ankle, with a bruise and significant swelling, what would you do?

    a) Immobilise the ankle and apply heat, until seen by medical services.

    b) Move the ankle gently, even if it hurts a little, to lubricate the joint.

    c) I wouldn't do anything, but I would take her to be seen by the medical services

    d) Immobilise the ankle and apply local cold, until seen by medical services.

    How confident are you in your answer to this question?

    a) Very uncertain. b) Somewhat certain. c) Certain. d) Very certain.

24. A person suffering from asthma tells you that they are having difficulty breathing and that they have not brought their rescue inhaler with them. What do you consider to be the most appropriate course of action?

    a) Sit her down quickly and reassure her.

    b) Lay her on her back and tell her to calm down.

    c) Ask if anyone has an inhaler, if they do, call the emergency services, and, while waiting, check if you can assist in administering a bronchodilator.

    d) Ask if anyone has an inhaler similar to yours and give it to her.

    How confident are you in your answer to this question?

    a) Very uncertain. b) Somewhat certain. c) Certain. d) Very certain.

25. In the case of a conscious person who has choked on a piece of meat, has his hands on his neck, and is unable to cough, indicate the most appropriate course of action:

    a) If the person loses consciousness, wait to perform CPR manoeuvres.

    b) Perform only back blows with the person leaned forward.

c) Encourage him/her to try to cough forcefully.

d) Stand behind him/her, perform five back blows and five Heimlich manoeuvres.

How confident are you in your answer to this question?

a) Very uncertain. b) Somewhat certain. c) Certain. d) Very certain.

26. For the current CPR protocol, please indicate the correct option and order:

a) Calm down, call for help, assess the injured, and do not intervene.

b) Call the emergency services, determine the level of consciousness, open the airway (see, hear and feel), do not perform chest compressions, and perform ventilations.

c) Determine level of consciousness, open airway (see, hear and feel), call emergency services, perform chest compressions, and perform ventilations.

d) Determine level of consciousness, call emergency services, open airway (see, hear and feel), perform ventilations, and perform chest compressions.

How confident are you in your answer to this question?

a) Very uncertain. b) Somewhat certain. c) Certain. d) Very certain.

27. In the case of a leg fracture where the broken bone is visible through the skin, what is the appropriate course of action, in addition to activating the emergency services?

a) Wrap the leg with sterile gauze pads.

b) Cover the wound with sterile gauze, and attach the affected leg to the other leg, with or without adding a brace (stick or board) to prevent them from moving.

c) Apply gentle pressure to try to reinsert the bone and immobilise the leg.

d) Apply localized cold therapy.

How confident are you in your answer to this question?

a) Very uncertain. b) Somewhat certain. c) Certain. d) Very certain.

28. You find a person on the ground in a public space. They are unconscious and not breathing (cardiac arrest), and you know there is an automated defibrillator (AED) in the vicinity. The most appropriate response is…

   a) After verifying that he/she is not breathing, I begin CPR, because I cannot use the AED as I am not specifically trained.

   b) I shout for help, verify that he is not breathing, call 911, get the AED, and follow the AED prompts.

   c) After verifying unconsciousness and that he is not breathing, I call 911; while someone goes to get the AED, I start CPR, and we follow the AED prompts.

   d) I shout and call for help, looking for qualified emergency personnel, without doing anything else until they show up.

   How confident are you in your answer to this question?

   a) Very uncertain. b) Somewhat certain. c) Certain. d) Very certain.

29. In the event of nosebleed (epistaxis), what should we do?

   a) Lean the head forward and compress the soft part of the nose against the nasal septum for 5 minutes.

   b) Lean the head backwards and compress the soft part of the nose against the nasal septum for 5 minutes.

   c) Lean the head forward and place gauze in the nasal passage without obstructing the nose.

   d) Place the head in a neutral position and blow the nose.

   How confident are you in your answer to this question?

   a) Very uncertain. b) Somewhat certain. c) Certain. d) Very certain.

30. In the event of heat exhaustion, what actions would you take?

   a) Move to a cool area and cover with a wet sheet or equivalent.

   b) Activate emergency services and lay the person on the ground with their legs elevated 30-40 centimetres.

   c) Move to a cool area, hydrate the person with an isotonic drink, and call the emergency services for transport to a hospital immediately.

d) Hydrate the person with water and continue the activity at a lower intensity.

How confident are you in your answer to this question?

a) Very uncertain. b) Somewhat certain. c) Certain. d) Very certain.

31. You are awakened by a prickling sensation in your right forearm. You discover that you have an unknown insect on your forearm. You discover that you have a spider on your forearm. Which of the following would be your attitude?

a) Remove the insect, clean the affected area, and put ice on it without direct skin contact

b) Remove the insect, scratch lightly, and try to remove the liquid by exerting pressure on the stinging point.

c) Remove the insect, do not cover the sting, and allow it to air out.

d) Remove the insect, clean the affected area, leave it to air, and do not scratch the itchy spot.

How confident are you in your answer to this question?

a) Very uncertain. b) Somewhat certain. c) Certain. d) Very certain.

32. If a person is sustained a head injury, reports neck pain, and is unable to move his or her legs, then....

a) We will leave the injured person at the place where he/she is located.

b) Take the injured person quickly to a hospital in our car.

c) Do not move the person, reassure them, call the emergency services so that they can immobilise them.

d) Immediately call the emergency services and observe that the person is breathing.

How confident are you in your answer to this question?

a) Very uncertain. b) Somewhat certain. c) Certain. d) Very certain.

33. When faced with a conscious person who presents a deep bleeding wound and dark red bleeding, what should you do?

a) Clean and disinfect with cotton wool and antiseptic, cover the wound so that it does not become infected with clean gauze.

b) Clean the wound, cover with clean gauze pads.

c) Clean the wound with water, apply compression with gauze, and, if it bleeds again, apply more gauze, without removing the first one.

d) Remove the foreign body, wash with water, and do not apply compression.

How confident are you in your answer to this question?

a) Very uncertain. b) Somewhat certain. c) Certain. d) Very certain.

34. In the case of a person with a seizure...

a) If it lasts more than 1 minute, it is considered an extreme emergency situation, so the emergency services should be notified quickly.

b) Let the person convulse, clear the area, and cushion their head with a jacket, pillow, or any other object.

c) When the seizure is over, place them in the recovery position.

d) Put something in his mouth to prevent him from biting his tongue.

How confident are you in your answer to this question?

a) Very uncertain. b) Somewhat certain. c) Certain. d) Very certain.

35. If a person is unconscious, breathing, and vomiting, the action to take is....

a) Get into the recovery position.

b) Sit the person with the body leaning slightly forward.

c) Do not touch the person unnecessarily, as we do not know if we can aggravate other problems.

d) Elevate the legs to raise blood pressure.

36. If a person has collapsed and is on the ground after a sudden fall, we approach and find that they are unconscious and breathing, what should we do?

a) Call the emergency services.

b) Put the person on his/her back to help them breathe, and elevate the legs.

c) Place him/her in the recovery position, call the emergency services, and periodically check that he/she is still responsive and breathing.

d) Perform chest compressions

How confident are you in your answer to this question?

a) Very uncertain. b) Somewhat certain. c) Certain. d) Very certain.

37. In the event of an adult cardiopulmonary arrest in which you are going to use the AED, you see that the adult pads are expired.

a) I still perform CPR manoeuvres with the AED even if they are out of date.

b) I only perform Basic CPR, as the AED is not effective with expired pads.

c) I perform CPR with AED but using the paediatric pads which are up to date.

d) I do nothing and wait for the emergency services to bring a suitable AED.

How confident are you in your answer to this question?

a) Very uncertain. b) Somewhat certain. c) Certain. d) Very certain.

Thank you for your help.
